# Compromised redox homeostasis, altered nitroso–redox balance, and therapeutic possibilities in atrial fibrillation

**DOI:** 10.1093/cvr/cvw012

**Published:** 2016-01-19

**Authors:** Jillian N. Simon, Klemen Ziberna, Barbara Casadei

**Affiliations:** Radcliffe Department of Medicine, Division of Cardiovascular Medicine, University of Oxford, Oxford, UK

**Keywords:** Hydrogen peroxide, Nitric oxide, Antioxidants, Subcellular localization, Arrhythmia, Heart

## Abstract

Although the initiation, development, and maintenance of atrial fibrillation (AF) have been linked to alterations in myocyte redox state, the field lacks a complete understanding of the impact these changes may have on cellular signalling, atrial electrophysiology, and disease progression. Recent studies demonstrate spatiotemporal changes in reactive oxygen species production shortly after the induction of AF in animal models with an uncoupling of nitric oxide synthase activity ensuing in the presence of long-standing persistent AF, ultimately leading to a major shift in nitroso–redox balance. However, it remains unclear which radical or non-radical species are primarily involved in the underlying mechanisms of AF or which proteins are targeted for redox modification. In most instances, only free radical oxygen species have been assessed; yet evidence from the redox signalling field suggests that non-radical species are more likely to regulate cellular processes. A wider appreciation for the distinction of these species and how both species may be involved in the development and maintenance of AF could impact treatment strategies. In this review, we summarize how redox second-messenger systems are regulated and discuss the recent evidence for alterations in redox regulation in the atrial myocardium in the presence of AF, while identifying some critical missing links. We also examine studies looking at antioxidants for the prevention and treatment of AF and propose alternative redox targets that may serve as superior therapeutic options for the treatment of AF.

## 1. Introduction

Atrial fibrillation (AF) has long been associated with atrial oxidative stress. Earlier studies pointed to irreversible oxidative modifications of proteins in patients with permanent AF,^1^ which has largely been attributed to an increased production of superoxide anion ($\left[{{\text{O}}_{2}}^{-}\right]$).^2–5^ We now better appreciate that several redox reactive oxygen species (ROS) and reactive nitrogen species (RNS) are altered in AF, both spatially and temporally, and that these biomolecules act as second messengers to promote both transient and sustained effects on the atria. Nevertheless, we have only scratched the surface in understanding the complexities of redox homeostatic regulation or how the network of redox-sensitive processes is altered in AF. In this review, we provide a discussion of where we are in our knowledge of altered redox homeostasis in the context of AF pathogenesis and what remains unknown. We begin by briefly reviewing some of the fundamental aspects of redox homeostasis, before focusing on recent studies that have revealed critical aspects of altered nitroso–redox balance in AF, while highlighting the considerable gaps in our knowledge. We will also discuss the attempts made thus far to treat AF using general antioxidant therapies and why these have remained largely unsuccessful and propose future therapeutic options that may prove more effective.

## 2. Reactive nitrogen and oxygen species as second messengers

### 2.1 Biologically relevant forms

**Figure 1:**
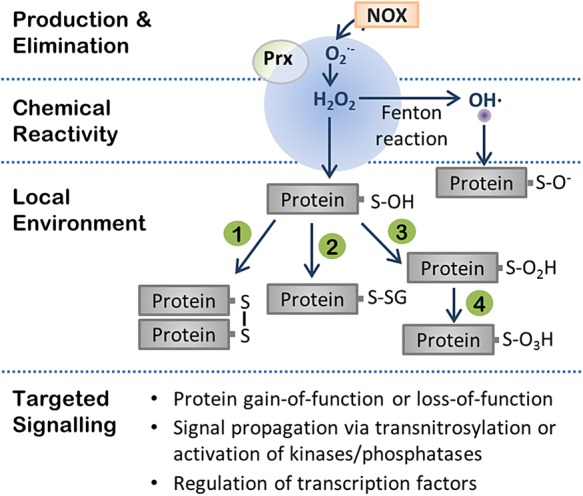
Regulation of redox second messenger systems. Production of $\left[{{\text{O}}_{2}}^{-}\right]$ by membrane-bound NADPH oxidase is rapidly dismutated to form the non-radical oxidant H_2_O_2_. The diffusion of H_2_O_2_ (shown in blue) is highly restricted in this microdomain due to the expression of Prx, which acts to rapidly reduce H_2_O_2_ to water. H_2_O_2_ may also undergo further reduction to OH· via Fenton reaction in the presence of reduced metals. Targeting of protein thiols by OH· leads to generation of a thiyl radical (SO^−^). In this schematic, the diffusion distance of OH· (in purple) is shown relative to the diffusion distance of H_2_O_2_, based on previous calculations,^18^ to demonstrate how target substrate oxidation would be restricted to intracellular targets with this radius. The local environment will also influence the type of thiol modification induced by the oxidant. In this example, H_2_O_2_ reaction with a cysteine thiolate promotes formation of sulphenic acid (SOH). Sulphenic acid can then further react to form a disulphide bond with another reactive cysteine thiolate in close proximity (Reaction 1). Alternatively, glutathione may also react with the sulphenic acid to form a mixed disulphide (*S*-glutathionylation; Reaction 2). Chronic ROS elevations in the face of compromised antioxidant defences may, instead, promote formation of irreversible modifications such as sulphinic acid (SO_2_H) (Reaction 3) and sulphonic acid (SO_3_H) (Reaction 4). The downstream consequence of such modifications can vary, including: gain or loss of function of the modified protein; further propagation of the signal via transnitrosylation or by oxidant-induced activation of signalling kinases or phosphatases; and regulation of gene transcription by redox-sensitive transcription factors.

Although free radicals were initially thought to be toxic cellular byproducts, we now appreciate their essential role in fine-tuning cellular function through redox-dependent signalling. Moreover, alterations in redox homeostasis have been implicated in a number of cardiovascular diseases.^6^ In most instances, indications of ‘oxidative stress’ have been correlated with pathological changes without much distinction between redox species. However, one must consider each redox species's biochemical profile to truly appreciate its biological impact. The ROS/RNS that have relevance in AF include the free radicals (one-electron oxidant) $[{{\text{O}}_{2}}^{-}],$ hydroxyl (OH·), and nitric oxide (NO) and non-radical (two-electron oxidant) species hydrogen peroxide (H_2_O_2_) and peroxynitrite (ONOO^−^). As illustrated for H_2_O_2_ in *Figure 1*, these redox biomolecules possess a complex regulatory network through which they alter cellular function.

### 2.2 Regulation of ROS/RNS production and elimination

Similar to all second messenger systems, the production and elimination of redox biomolecules are tightly regulated in a spatial and temporal manner, which is essential for promoting signalling selectivity. However, one must also appreciate that formation of ROS/RNS in one compartment may promote formation of ROS/RNS in other compartments either directly, as is the case in formation of ONOO^−^ from $\left[{{\text{O}}_{2}}^{-}\right]$ and NO, or indirectly, as has been shown for NOX2-mediated activation of mitochondrial ROS.^7^ In fact, it is most likely that any chronic production of ROS occurs over a continuum in which sustained alterations in the nitroso–redox balance promote further changes in ROS/RNS formation at other subcellular compartments leading to what is commonly referred to as oxidative stress.^8^

Spatial regulation of ROS/RNS in the heart is accomplished by confinement of the ROS/RNS-generating enzymes, along with the associated antioxidant systems, to the mitochondria, the sarcoplasmic reticulum (SR), and the sarcolemmal membrane. In the mitochondria, $[{{\text{O}}_{2}}^{-}],$ H_2_O_2_, and OH· are generated as natural byproducts of inefficient oxidative phosphorylation.^9^ Owing to the high expression level of mitochondrial superoxide dismutase (SOD1, intermembrane space; SOD2, matrix), most of the $\left[{{\text{O}}_{2}}^{-}\right]$ is converted to H_2_O_2_. This H_2_O_2_ is, in turn, reduced by the thioredoxin 2 (Trx2) antioxidant system^10–12^—which includes Trx2, thioredoxin reductase 2, and peroxiredoxins 3 and 5 (Prx3 and Prx5)—or by glutathione peroxidases (Gpx1 and Gpx4)^13,14^ to form water. Besides acting on mitochondrial proteins directly, the generated H_2_O_2_ can also readily diffuse into the cytosol where it can activate redox-dependent signalling. In addition, during states of enhanced mitochondrial ROS production, H_2_O_2_ can undergo further reduction within the mitochondria, through Fenton reaction catalysed by reduced metals, to form the highly reactive OH·.

Localized production of $\left[{{\text{O}}_{2}}^{-}\right]$ and H_2_O_2_ also occurs at the SR and sarcolemmal membrane, as does the production of NO. Both xanthine oxidoreductase (XOR) and neuronal NO synthase (nNOS or NOS1) are localized to the SR where they participate in the formation of $\left[{{\text{O}}_{2}}^{-}\right]$ and NO, respectively.^15^ Moreover, these two enzymes appear to be tightly coupled such that nNOS acts to attenuate XOR activity and, as a consequence, maintain low levels of $\left[{{\text{O}}_{2}}^{-}\right]$ under normal conditions.^15,16^ Within the sarcolemmal membrane, NADPH oxidase 2 (NOX2) is the primary source of $\left[{{\text{O}}_{2}}^{-}\right]$ production in human atrial myocytes,^2,3^ with clustering thought to occur within caveolae.^17^ Upon activation, the NOX2 subunits assemble and function to reduce oxygen to $[{{\text{O}}_{2}}^{-}].$ The current convention is that, topographically, NOX2 is positioned within the membrane such that $\left[{{\text{O}}_{2}}^{-}\right]$ is generated outside the cell and rapidly dismutated, by extracellular SOD, to form H_2_O_2_.^18^ Therefore, any downstream effects of NOX-generated $\left[{{\text{O}}_{2}}^{-}\right]$ are likely to be mediated by H_2_O_2_ and could occur either extracellularly or by transport of H_2_O_2_ intracellularly.

Scavenging of the $\left[{{\text{O}}_{2}}^{-}\right]$ produced in both the SR and the sarcolemmal membrane occurs either through sequestering by nearby NO, at a diffusion-limited rate, to form the less reactive ONOO^−^ or by dismutation by the cytosolic isoform of SOD (Cu/ZnSOD; SOD1) to form H_2_O_2_. The generated ONOO^−^ is further neutralized by NO, CO_2_, uric acid, or reduced glutathione (GSH) while H_2_O_2_ is reduced by Prx (both Prx1 and Prx2) and by the antioxidant enzymes associated with GSH metabolism. It should also be noted that not all antioxidants will reduce a given oxidant with the same efficiency. For example, Prx is one of the major buffers for H_2_O_2_ in the cell, as GSH reacts too slowly with H_2_O_2_ to provide adequate buffering.^19^ These preferences are mainly driven, however, by whether the oxidant is a radical or non-radical species.

Both endothelial NOS (eNOS or NOS3) and nNOS are constitutively expressed in the myocardium where they localize to the sarcolemmal and SR membrane, although their subcellular localization can be altered under various stimuli.^20^ eNOS is found to be associated with caveolae where it binds caveolin-3 in the inactive state.^21^ Conversely, nNOS is anchored to the sarcolemmal membrane through association with a macromolecular complex including syntrophin and dystrophin^22^ and to the SR, in the proximity of XOR, as already mentioned. In the coupled state, NOSs produce NO, which is then buffered by the local presence of thiols and myoglobin. However, in an uncoupled state (via *S*-glutathionylation^23^ or altered cofactor/substrate availability^24,25^), NOS leads, instead, to the production of $[{{\text{O}}_{2}}^{-}].$

### 2.3 Chemical reactivity and the influence of the local environment

Apart for subcellular compartmentalization of ROS/RNS production and elimination, signalling selectivity by redox biomolecules is also influenced by the preferred redox chemistry (e.g. one- or two-electron oxidations) of each species, the half-life of the redox biomolecule, as well as the local redox potential (i.e. chemical environment). These biochemical properties will greatly influence the number of downstream targets, the distance of those targets from the primary source of ROS/RNS generation, and the type of modification a given ROS/RNS will yield. In most instances, due to the short half-life of redox biomolecules, oxidative reactions are often targeted to reactive residues on proteins in close proximity to the source of ROS/RNS production. This is particularly true for highly reactive species such as OH· whose half-life is 10^−9^ s, whereas more stable species such as H_2_O_2_ (half-life of 1 ms) can diffuse away from their source.^26^

ROS non-radical species are also influenced by both reduction potential and reaction kinetics (or reactivity). For example, although H_2_O_2_ has a high reduction potential relative to other species (*E*_o_ = 1.77 V for H_2_O_2_ vs. *E*_o_ = –0.80 V for NO), it has the slowest reactivity owing to its high activation energy.^18^ Given that target oxidation depends both on reactivity and on the localized concentration of an oxidant, relatively high concentrations of H_2_O_2_ (µM) would be necessary to facilitate protein oxidation and downstream signalling. This would also be true for radicals which have low reduction potentials, such as NO; whereas species such as OH· that have high reduction potentials (*E*_o_= 2.31 V) readily oxidize thiols when present in low concentrations.

Given the poor reactivity of H_2_O_2_, it would be unlikely for the peroxide to modify protein thiols, and therefore mediate intracellular signalling, under states of low production in which majority of the H_2_O_2_ is scavenged by Prx. However, when localized peroxide concentrations increase, H_2_O_2_ is able to restrict Prx2 function by hyperoxidation of the enzyme, rendering it inactive. This so-called ‘floodgate’ mechanism^27^ allows for an enhanced accumulation of H_2_O_2_, as well as increasing the probability for H_2_O_2_ to react with other thiol targets and facilitate signalling.

### 2.4 Downstream targets of ROS/RNS

Oxidants promote their biological effect by reversibly modifying proteins primarily on the sulphur-containing amino acids cysteine and methionine, with reactivity of the cysteine thiolate (S^−^) being approximately four-fold faster than that of methionine.^28^ The types of reversible oxidative cysteine thiol modifications include *S*-nitrosylation, *S*-sulphenylation (SOH), or disulphide bond formation, either between two cysteine residues or with glutathione (*S*-glutathionylation). Functionally, these modifications act as a regulatory redox switch to induce either gain or loss of function of the modified protein, although they may also serve to protect a protein from irreversible oxidative damage.^29^ More recent evidence demonstrates that redox biomolecules may also propagate signals to other subcellular compartments through targeting of redox-sensitive transcription factors, by protein transnitrosylation (i.e. transfer of an NO modification from one protein to another), or through targeting of other signalling molecules, such as kinases and phosphatases. Indeed, in the context of AF, angiotensin-II-induced oxidation of Ca^2+^/calmodulin-dependent protein kinase 2 (CaMKII) was shown to result in increased SR Ca^2+^ leak through ryanodine receptor, leading to an increased susceptibility to AF in mice.^30^

## 3. Spatiotemporal alterations of redox homeostasis in AF

Despite an expanding amount of evidence implicating oxidative stress to AF, there are surprisingly little mechanistic data linking changes in redox homeostasis to the associated cellular dysfunction. In addition, as we hope that the previous section has highlighted, to generalize oxidative stress as one single biological entity is counterproductive for the understanding of both the mechanism of action by various redox biomolecules and their role in the disease. With this in mind, we proceed by considering what the available data tells us about altered redox homeostasis in AF pathogenesis and what remains to be considered.

### 3.1 Temporal and spatial alterations in redox homeostasis associated with AF

It is well accepted that AF is associated with elevations in atrial ROS production.^2,4,31–33^ This is true both for the development of post-operative AF and for paroxysmal or long-standing persistent AF, although the sources of oxidants differ with disease progression.^2,32^ In the case of post-operative and paroxysmal AF, NOX2-derived $\left[{{\text{O}}_{2}}^{-}\right]$ has been shown to be the major source of ROS production.^2,4,32^ This NOX2-mediated increase in myocardial $\left[{{\text{O}}_{2}}^{-}\right]$ at the time of surgery was shown to be predictive of AF development in the post-operative period.^31^ Moreover, mice with cardiac-specific overexpression of Rac-1, a necessary activator for NOX2, develop AF spontaneously,^34^ collectively supporting a role for myocardial oxidase systems in determining an atrial substrate for the new onset of AF. Others have also shown intracellular formation of $\left[{{\text{O}}_{2}}^{-}\right]$ by XOR in animal models of short-term tachypacing-induced AF^4,35,36^; however, the relevance of myocardial XOR-derived $\left[{{\text{O}}_{2}}^{-}\right]$ in humans is unclear.^2^ Beyond this, we know little more about alterations in redox homeostasis in the early stages of AF. In general, NOS activity appears to be coupled in patients who go on to develop AF post-operatively^32^ and in animal models of tachypacing-induced AF,^32^ although it is unclear whether localization of NOS is altered at this early time-point.

Both the sources of ROS production and the status of NOS seem to change over time in AF. This was demonstrated using a goat model of AF over the course of 6 months.^32^ In this case, production of $\left[{{\text{O}}_{2}}^{-}\right]$ by NOX was shown to be increased in the left atrium after 2 weeks of AF, whereas at the 6-month time-point, the main sources of increased $\left[{{\text{O}}_{2}}^{-}\right]$ production were the mitochondria and uncoupled NOS. Elevations in $\left[{{\text{O}}_{2}}^{-}\right]$ from both mitochondrial sources and NOS uncoupling were further confirmed in the right atrial appendage (RAA) of patients with permanent AF. This finding suggests that the pathological continuum of AF likely influences subcellular compartmentalization of ROS/RNS. Indeed, the same study showed increased production of $\left[{{\text{O}}_{2}}^{-}\right]$ from mitochondria and uncoupled NOS in goats with structural remodelling (secondary to AV node block) in the absence of AF, implying that the production of ROS by these sources is likely driven by atrial structural remodelling rather than by the arrhythmia itself. Nevertheless, it is still not clear whether early and sustained changes in redox homeostasis during AF may promote subsequent induction of ROS from other sources, which could be important for advancing the disease, particularly given that mitochondrial ROS and NOS uncoupling are well known to be induced by other ROS sources*.*^7,24^

It is worth highlighting that in the abovementioned study, only $\left[{{\text{O}}_{2}}^{-}\right]$ levels were measured over the time-course and, therefore, interpretation is limited to changes in $\left[{{\text{O}}_{2}}^{-}\right]$ production without consideration for alterations in other ROS. Put another way, it is plausible that oxidants such as H_2_O_2_ could be increased as a consequence of blunted degradation without changes in formation, via dismutated $[{{\text{O}}_{2}}^{-}].$ Indeed, Prx3 (the mitochondrial isoform of Prx) was shown to be reduced in canine left atria after 2 weeks of ventricular tachypacing.^37^ Moreover, the detection of Prx3 using two-dimensional gel electrophoresis showed a shift in the isoelectric point towards the acidic region, indicative of post-translational modification following tachypacing. This isoelectric shift may indicate Prx3 oxidation, as has been shown by others,^38^ or phosphorylation, either of which is known to inactivate the enzyme and promote sustained increases in H_2_O_2_.^27,39^

**Figure 2:**
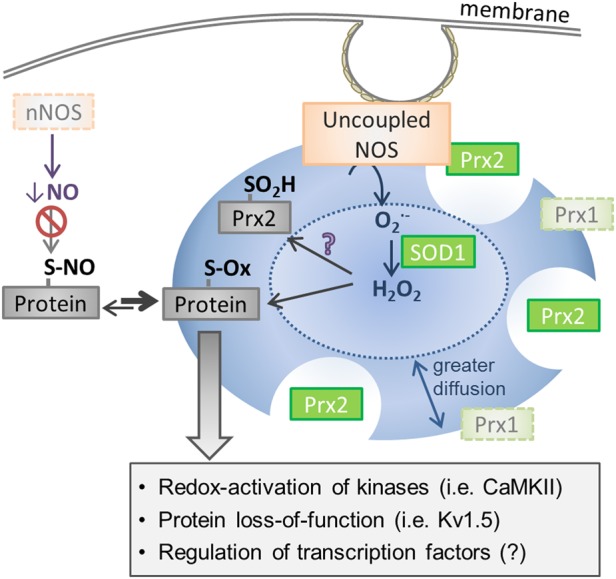
Known and predicted changes in compartmentalized redox homeostasis in permanent AF. The primary sources of oxidant generation in permanent AF include ROS produced from uncoupled NOS (within the membrane and/or cytosolic compartment) and the mitochondrial electron transport chain. The $\left[{{\text{O}}_{2}}^{-}\right]$ produced from uncoupled NOS is dismutated to H_2_O_2_ by SOD1, whose expression is increased in permanent AF, most likely to compensate for the increased $\left[{{\text{O}}_{2}}^{-}\right]$ production. In addition to increased production of H_2_O_2_, elimination of H_2_O_2_ is compromised by loss of cytosolic Prx1. This further increases localized H_2_O_2_ concentration and likely the diffusion distance for H_2_O_2_. Consequently, these alterations in local redox homeostasis would promote oxidation of nearby thiols, which may include Prx2 to promote sustained H_2_O_2_ levels, leading to a number of various downstream effects. In addition, NO production is reduced in permanent AF owing to both NOS uncoupling and loss of nNOS. Both the increase in ROS and the reduction in RNS negatively shift the nitroso–redox balance in favour of protein oxidation, which is thought to contribute to the electrical changes associated with AF.

Looking at the available data regarding altered redox homeostasis in long-standing persistent AF, one can develop some reasonable hypotheses regarding subcellular compartmentalization and nitroso–redox balance (*Figure 2*). First, the major redox biomolecule in permanent AF is likely to be H_2_O_2_ and not $\left[{{\text{O}}_{2}}^{-}\right]$, at least in certain compartments of the myocyte. Upregulation of the cytosolic isoform of SOD (SOD1) has been shown to occur in AF patients^40^ and would act to rapidly reduce all generated (cytosolic) $\left[{{\text{O}}_{2}}^{-}\right]$ to H_2_O_2_. In addition, one might expect a longer half-life for the generated H_2_O_2_, as Prx1 is known to be reduced in permanent AF,^41,42^ and would therefore impair rapid clearance of H_2_O_2_ and, perhaps, even increase its diffusion distance. Given the low p*K*_a_ of protein thiols,^18^ this large, sustained production of H_2_O_2_ is probably necessary for it to act as a second messenger to alter cellular function in AF. Indeed, an increase in global sulphenylation—the product of the reaction between H_2_O_2_ and a protein thiol—in RAA from patients with permanent AF has been reported.^43^ One might also expect the elevated H_2_O_2_ concentrations to promote hyperoxidation and inactivation of Prx2, via the floodgate mechanism, resulting in chronic peroxide accumulation, although this has not been directly assessed in AF.

There is also evidence in permanent AF for irreversible oxidative modifications of the sarcomeric proteins.^1^ The presence of both carbonylation and nitration has been observed, suggesting that some of the $\left[{{\text{O}}_{2}}^{-}\right]$ produced preferentially reacts with nearby NO to rapidly form ONOO^−^. Furthermore, because ONOO^−^ is highly reactive, its formation undoubtedly occurs in close proximity to the myofilament proteins which are oxidized. Although these modifications are most often thought to serve as biomarkers for redox stress as opposed to mediating any regulated adaptation of the cell to increased ROS/RNS production, they can lead to loss of protein function, which will likely have a biological impact. Indeed, the authors found that nitration, but not carbonylation, of the myofibrillar form of creatine kinase (MM-CK) negatively correlated with MM-CK function in myofibrillar tissue from AF patients.^1^

Finally, NOS isoforms appear to be differentially altered in permanent AF. Although NOS uncoupling, possibly of both eNOS and inducible NOS, has been shown in several studies,^32,44,45^ there is recent evidence for loss of nNOS in the RAA of patients with permanent AF, which could be recovered by prevention of miR-31 binding to the nNOS 3′-UTR.^46^ Interestingly, selective inhibition of nNOS in atrial myocytes isolated from patients in sinus rhythm (SR) mimicked the action potential (AP) shortening observed in AF, but had no effect in atrial myocytes isolated from patients with AF. Moreover, inhibition of miR-31 in AF myocytes normalized both nNOS protein levels and AP changes, suggesting that nNOS plays a key role in the AF-induced electrical remodelling. This point probably best highlights the emerging concept that an altered nitroso–redox balance is more critical than oxidative stress *per se* in AF's underlying mechanisms.

### 3.2 Shifting the nitroso–redox balance in AF

This idea of maintaining NO and ROS in balance (e.g. nitroso–redox balance), which has developed in recent times, is, to some degree, unsurprising, given the clear overlap in their production, functionality, and breakdown. In several instances, shifting the nitroso–redox balance in favour of increased NO production, particularly by enhancing nNOS activity, has proved to be beneficial to the heart.^47–49^ The abovementioned study by Reilly *et al*.^46^ suggests that this may also be true in the context of persistent AF. Nevertheless, the mechanism by which cardioprotection occurs is unclear, although some studies suggest that this may occur via protection from deleterious oxidative modification (both locally^29,47^ and at distal sites^50^) or indirectly through regulation of local H_2_O_2_ concentrations.^51,52^

Nevertheless, very little is known regarding the mechanistic link between the nitroso–redox imbalance and electrophysiological impairment in AF. Although a number of ion channels and transporters have been shown to be redox-sensitive (see Wolke *et al*.^53^ for a recent review), only a few studies have looked at this in the context of AF. It has been shown in the left atrial appendage of patients in long-standing AF that hyper-nitrosylation of the l-type Ca^2+^ channel, within the α_1c_ subunit, occurs and is inversely related to the total cellular glutathione content.^54^ In addition, the *I*_Ca,L_ was reduced in left atrial myocytes from these patients, an effect mediated by α_1c_-nitrosylation.^55^ Nevertheless, others have failed to show α_1c_-nitrosylation in RAA from patients with long-standing AF,^56^ suggesting that alterations in nitroso–redox balance may differ between sides.^32^

Kv1.5 (*I*_Kur_) is also well known to be redox-sensitive, with both sulphenylation and nitrosylation reported.^43,57^ An increase in sulphenylation of Kv1.5 was identified in the RAA of patients with permanent AF.^43^ This modification was shown to cause a reduction in the surface expression of the channel by promoting internalization. Conversely, nitrosylation of Kv1.5 has been shown to inhibit *I*_Kur_ without altering the channel's expression or localization.^57^ Interestingly, it has been proposed that NO may act to tonically inhibit *I*_Kur_ under physiological conditions, while loss of NO in long-standing AF—either through NOS uncoupling or loss of nNOS—would result in the release of this tonic inhibition, leading to an increase in *I*_Kur_ and the promotion of arrhythmias.^58^ One might also speculate that loss of the nitrosothiol on Kv1.5 is what allows for sulphenylation of the reactive thiolate in permanent AF—an argument that is supported by recent investigations.^29,47^ Thus, investigating how shifts in the nitroso–redox balance can alter protein redox modifications and contribute to cellular dysfunction in AF may prove informative.

Beyond direct effects on myocardial electrical properties, an altered redox environment may also promote adverse structural remodelling, providing a substrate for AF sustainment. In particular, inflammation and fibrosis have been linked to changes in the redox system, through myeloperoxidase, with implications on atrial conduction and AF pathophysiology.^59,60^ The reader is referred to recent reviews,^61,62^ which offer a more in-depth discussion of these links. Likewise, atrial strain may also promote ROS release,^32,63^ which could contribute to AF pathogenesis, although evidence for this is currently lacking.

## 4. Therapeutic approaches targeting redox balance

In addition to its relevance in AF, altered redox homeostasis is considered to be an integral component in the pathogenesis of cardiovascular disease as a whole. Tackling redox imbalance with antioxidants has thus been tested under multiple different conditions with strong backing from experimental models of disease; however, results in humans have been disappointing. Antioxidant vitamins as a whole or individually did not reduce mortality, cardiovascular death, incidences of angina pectoris, myocardial infarction, or stroke.^64–66^ Furthermore, there was no benefit of using those antioxidant vitamins for either primary or secondary prevention of cardiovascular diseases, suggesting that antioxidants have no effect on reducing global mortality or morbidity. Nevertheless, it still remains unclear whether antioxidants may be beneficial in specific diseases in which alterations in redox homeostasis play a more causative role, such as AF.

### 4.1 General antioxidants for prevention of post-operative AF

The first clinical study on antioxidants in AF by Carnes *et al*.^5^ assessed the utility of ascorbic acid (vitamin C) for the prevention of post-operative AF, showing a 50% reduction in the incidence of post-operative AF in the ascorbic acid group when compared with the control group. However, follow-up studies using very similar dosing regimen, but enrolling more patients (100–185 per study) and applying more rigorous protocols (randomized double-blind placebo-controlled clinical trials), showed mixed results.^67–69^ Results using N-acetylcysteine, another widely studied general antioxidant, for the prevention of post-operative AF have also generally yielded negative results.^70–75^ One of the main criticisms of these trials, however, is that vast majority of them were severely underpowered to reliably detect the expected differences in the incidence of post-operative AF. In addition, these antioxidants were tested under the assumption that they neutralize free radicals, as has been demonstrated *in vitro*.^76,77^ However, we now better appreciate that in the *in vivo* setting, such antioxidants do not reach sufficient, localized concentrations to overcome kinetic limitations and allow for scavenging of highly reactive free radical species.^78^ The only exception to this is α-tocopherol (vitamin E), which has been shown to directly inhibit lipid peroxidation.^79^

Instead of attempting to scavenge oxidants, a strategy aimed at preventing the production of radicals or non-radicals would be more advantageous. This could be accomplished either through inhibition of oxidant-producing enzymes (such as NOX2 for $\left[{{\text{O}}_{2}}^{-}\right]$) or through enhancement of endogenous antioxidant enzymes to catalyse the reduction of non-radicals and thereby prevent damaging free radical formation (such as OH· from H_2_O_2_). Indeed, there is evidence that this occurs when a combinatorial antioxidant regimen (ascorbic acid, α-tocopherol, and polyunsaturated fatty acids) is used, and small trials thus far have shown positive results for use of this regimen to treat post-operative AF.^80–82^ Of particular interest is work by Rodrigo *et al.*,^82^ in which the study endpoints were not only the incidence of post-operative AF, but also the effectiveness of the treatment regimen to improve biomarkers of impaired redox balance. In this small study, the incidence of post-operative AF was decreased when compared with the placebo group [10 cases (9.7%) in the supplemented group vs. 32 cases (32%) in the placebo group]. Furthermore, levels of malondialdehyde (a product of lipid peroxidation used as a biomarker of oxidative stress) in plasma and RAA were significantly decreased in the supplemented group when compared with the placebo group. They also observed higher activity for catalase, SOD and Gpx, along with decreased protein levels and gene expression of NOX2 (p47-phox subunit). Interestingly, although not noted by the authors, the observed changes in these antioxidant enzymes are all expected to occur upon activation of phase II antioxidant defences.^83^ Activation of this intrinsic signalling cascade, which is part of the cell's natural response to chronic oxidant elevation, has indeed been proposed as one of the primary mechanisms by which some antioxidants effectively work.^78^ Therefore, this combinatorial antioxidant regimen may offer a distinct mechanism (e.g. directly targeting enzymatic oxidant production and elimination) through which it exerts its beneficial effects. Furthermore, following radical scavenging by α-tocopherol, the α-tocopheroxyl radical can be recycled back to the non-radical form by ascorbate.^84^ Treating with these two antioxidants in combination, therefore, may help to maintain antioxidant levels and allow for more sustained effects. Although mechanistically promising, larger clinical trials are needed before we will reach a final verdict on the effectiveness of a combination of antioxidants for post-operative AF.

Statins, which attenuate [O_2_^−^] production by inhibiting Rac1 GTPase and decreasing NOX2 subunit expression,^32,85^ have also been tested as a potential therapy for the prevention of post-operative AF. Unfortunately, despite strong evidence from basic research and animal models,^61^ as well as positive results from initial smaller clinical trials (40–200 patients per study),^86^ a recently reported large-scale clinical trial assessing perioperative statin therapy in cardiac surgery (STICS trial) clearly demonstrated that statins do not prevent post-operative AF.^87^ Results of this study have implications not only for clinical management of AF, but also raise the question of whether redox imbalance is causative of post-operative AF or merely correlated with its development.

### 4.2 General antioxidants for secondary prevention of AF

Majority of the clinical trials evaluating the effect of antioxidants in the context of AF focused on preventing post-operative AF, primarily because post-operative AF was thought to have a more clearly defined pathological target (e.g. inflammation and increased $\left[{{\text{O}}_{2}}^{-}\right]$ production by NOX2) for application of general antioxidants. Secondary prevention of AF, in contrast, must attempt to reverse, or at least slowdown, atrial tissue remodelling, which is associated with an array of pathological mechanisms, including modification of ion channels, fibrosis, inflammation, metabolic alterations, and nitroso–redox imbalance.^88^ In particular, with regard to nitroso–redox imbalance, the redox status is likely to be differentially shifted depending on the subcellular compartment (e.g. mitochondria vs. sarcolemmal membrane), which, in turn, will have distinct functional effects (e.g. metabolic alterations vs. modification of ion channels). General antioxidants, however, do not target specific locations and are, instead, diffusely spread across the cells. Moreover, targeting of ROS by general antioxidants does not address the loss of NO bioavailability, which occurs in long-standing persistent AF.^32,46^ Given these limitations, it is not surprising that clinical trials with general antioxidants for the secondary prevention of AF have not shown favourable results.^89–92^

Statins have also been tested for secondary prevention of AF. Several double-blind placebo-controlled studies (64–222 patients per study) comparing the AF recurrence in statins vs. placebo groups in patients with paroxysmal or persistent AF have generally resulted in negative results.^93–95^ On the whole, it is surprising that the lipid-lowering effects of statins, and the documented reduction in ischaemic events, have no impact on the prevalence of AF. However, looking from the redox perspective, the lack of effect may be explained by the fact that NOX2 is not a major source of oxidant generation in persistent AF, nor are statins likely to correct the nitroso–redox imbalance which occurs in the later stages of AF pathogenesis. Therefore, if compromised redox homeostasis is fundamental to AF sustainment, it is less surprising that statin therapy is ineffective.

### 4.3 New drugs targeting redox homeostasis

As highlighted earlier, the idea that general antioxidants may act as scavengers of free radicals is unlikely. Even so, these antioxidants favourably target radical oxidant species, which participate in one-electron transfer reactions.^96^ These radical species are more likely to promote macromolecular damage as opposed to non-radicals, which participate to a greater extent in the regulation of cellular processes. In addition, their bioavailability is uniformly distributed across tissues, such that their concentration within microdomains would not reach levels sufficient to prevent oxidative damage during localized intensive bursts of ROS/RNS.^97^

To overcome these limitations, future antioxidant drug design would benefit from focusing more on specific antioxidants that either target the sources of ROS/RNS production directly (e.g. NOS re-couplers or inhibitors of XOR) or enhance the expression of endogenous antioxidant defences. There may also be therapeutic potential for agents that act as a concentrated ROS scavenger in a very specific location (e.g. mitochondrial ROS scavengers), or which pharmacologically modify downstream effectors of oxidant signalling (e.g. CaMKII inhibitors). In addition, compounds that act to restore nitroso–redox balance, particularly in the case of long-standing persistent AF (e.g. NOS recouplers), should be considered. *Table 1* summarizes the current stage of development of some promising and emerging new antioxidants.

**Table 1:** Emerging new antioxidant treatments for AF

Target	Pharmacological agents	Pre-clinical studies
Nitric oxide synthases (NOSs)	Tetrahydrobiopterin (BH4)	BH4 treatment decreased the AF inducibility in a canine tachypacing model (0/9 vs. 6/12 in placebo group)^45^
NADPH oxidase	Apocynin	Apocynin decreased superoxide production in human RAA of patients with AF by 81%.^2^ Apocynin treatment decreased the AF inducibility in a rabbit tachypacing model (17 vs. 67% in the placebo group)^98^
Xanthine oxidase	Oxypurinol	Oxypurinol decreased superoxide production in LA (by 85%) in the pig tachypacing model of AF.^4^ Oxypurinol did not change superoxide production in human RAA of patients with AF^2^
	Allopurinol	Allopurinol treatment decreased the duration of AF in a canine tachypacing model (0.6 vs. 173 s in the placebo group)
CaMKII	KN-93	KN-93 treatment decreased the arrhythmic phenotype in NOX4 overexpressed zebrafish hearts^99^
Mitochondrial ROS production	mitoTEMPO	mitoTEMPO treatment decreased sudden cardiac deaths in the cardiac renin–angiotensin system activation model of spontaneous ventricular arrhythmias (18 vs. 74% in the placebo group)^100^

AF, atrial fibrillation; BH4, tetrahydrobiopterin; NOS, nitric oxide synthase; NOX, NADPH oxidase; RAA, right atrial appendage; SR, sinus rhythm.

## 5. Conclusions and future perspectives

The evolution in our understanding of regulatory processes, which dictate redox homeostasis and the biochemical properties inherent to both radical and non-radical oxidants, has expanded greatly in recent times. Appreciation for compartmentalization of ROS/RNS production and elimination, as well as oxidant reactivity, has allowed for a better understanding of potential consequences and how this might relate to disease. In addition, studies have provided a general picture of changes in ROS/RNS compartmentalization over the duration of the disease, which is certain to dictate cellular targets and the downstream consequences. Recent findings also support the idea that alterations in the nitroso–redox balance are, perhaps, more important in mediating dysfunction than increases in ROS alone. Nevertheless, considerable gaps in our knowledge remain.

Progress towards understanding redox imbalance as a mechanism underlying AF, or how to best target these processes therapeutically, will not move sufficiently forward until we attempt to better appreciate the fundamentals of redox homeostasis or how the system as a whole is augmented in AF. Evidence in the redox signalling field suggests that two-electron (non-radical) transfer reactions are in all likelihood the redox pathway by which cellular processes are regulated; however, antioxidant therapies used thus far have attempted to target radical species without consideration for elevations in non-radical production, which are much more likely to be relevant in AF pathogenesis, or disease in general. These flaws have cast a shadow on the application of antioxidants for the treatment of cardiovascular disease, despite compelling evidence that compromised redox homeostasis is central to several pathologies. We propose that the design and application of antioxidant compounds which reduce formation of ROS while enhancing localized antioxidant buffering are worth testing in AF, as are those which address nitroso–redox balance.

## Funding

The authors are funded and supported by the British Heart Foundation (RG/11/15/29375, CH/12/3/29609), a Foundation Leducq Network of Excellence (08CVD01), the European Union 7th Framework Programme under Grant Agreement no. 261057 (EUTRAF), and a UK Medical Research Council scholarship to K.Z. Funding to pay the Open Access publication charges for this article was provided by the British Heart Foundation.
